# Quality assurance in generative AI-mediated education: a bibliometric and scoping review

**DOI:** 10.3389/frai.2026.1844517

**Published:** 2026-07-20

**Authors:** Daysi Yuliana López-Acosta, Silvia Rosa Pacheco-Mendoza, Domingo José Alvarado-Jaramillo, Patricio Rafael Zurita-Flores

**Affiliations:** State University of Milagro, Milagro, Ecuador

**Keywords:** academic integrity, artificial intelligence ethics, artificial intelligence in education, digital transformation in education, educational quality assurance

## Abstract

The rapid advancement of generative artificial intelligence has introduced transformative opportunities and critical challenges for quality assurance in educational settings. This study aims to systematically map the scientific landscape on quality assurance in education mediated by generative artificial intelligence, identifying predominant methodological approaches, conceptual frameworks, and emerging research gaps. A bibliometric and scoping review was conducted following PRISMA-ScR guidance and integrating descriptive bibliometric analysis with qualitative thematic mapping. The analysis was based on 482 documents retrieved from Scopus and Web of Science, covering the period 2022–2026. The data were analyzed using a combined approach that integrates descriptive bibliometric analysis and qualitative content analysis of the abstracts. The results show a predominance of review studies, indicating a phase of conceptual consolidation in the field. There is also an increase in quantitative and experimental studies, reflecting a transition toward empirical validation of the use of generative artificial intelligence in education. From a conceptual perspective, the literature is primarily oriented toward quality assessment and ethical implications, particularly concerning biases, transparency, and responsible use of AI. In contrast, pedagogical frameworks and academic integrity show limited development. These findings highlight a structural tension between technological innovation and the need to ensure educational quality, ethical governance, and academic integrity. The need to develop integrative frameworks that articulate pedagogical, technological, and normative dimensions is emphasized. This study contributes to the understanding of an emerging field and provides relevant inputs for researchers, educators, and educational policymakers interested in the responsible implementation of generative artificial intelligence.

## 1 Introduction

The accelerated development of generative artificial intelligence is significantly transforming educational systems worldwide, introducing new forms of interaction, content production, and knowledge construction (Al Naqbi et al., 2025). Tools based on large-scale language models, such as ChatGPT and similar systems, have been rapidly adopted in academic contexts, facilitating the automated generation of texts, personalized feedback, and adaptive learning experiences (Allaithy and Zaki, 2025; Alnsour et al., 2025). However, alongside these opportunities, substantial challenges arise related to educational quality assurance, academic integrity, and ethical governance.

In recent years, the incorporation of artificial intelligence in education has evolved from rule-based intelligent tutoring systems to generative models capable of producing content with a high degree of similarity to human language (Cordón García, 2023). This transition not only represents a technological advance but also a paradigm shift in the way knowledge is produced, evaluated, and validated within educational institutions. In this context, traditional quality assurance mechanisms—such as evaluation systems, academic standards, and accreditation processes—are challenged by the dynamic, complex, and often opaque nature of generative artificial intelligence systems.

One of the main debates emerging in this field concerns the tension between innovation and control. On the one hand, generative artificial intelligence offers opportunities to improve efficiency, accessibility, and personalization of learning (Bahroun et al., 2023; Francis et al., 2025; Wang et al., 2024). On the other hand, it poses risks associated with the generation of inaccurate information, algorithmic biases, lack of transparency, and potential impacts on academic integrity (Giannakos et al., 2025; Preiksaitis and Rose, 2023). This duality necessitates rethinking existing quality assurance frameworks to ensure their relevance in educational environments mediated by artificial intelligence.

Despite the growing volume of literature on artificial intelligence in education, research specifically focused on quality assurance in the context of generative artificial intelligence remains fragmented and heterogeneous. Many studies address isolated dimensions of the phenomenon, such as ethical implications, assessment practices, academic integrity, pedagogical affordances, or technological reliability, without offering an integrated view that articulates these dimensions within a quality assurance perspective (Ali et al., 2024; Giannakos et al., 2025; Wang et al., 2024; Yan et al., 2024; Nguyen et al., 2023).

In this sense, it is necessary to conduct a systematic analysis that maps the existing scientific production, identifies trends, methodological approaches, and conceptual frameworks, as well as detects gaps in the research. Scoping reviews constitute a suitable methodological tool for this purpose, as they allow the exploration of broad and emerging fields, facilitating the identification of opportunities for future research.

Previous reviews have examined artificial intelligence in education, ChatGPT, academic integrity, assessment practices, AI ethics, and generative AI in higher education from different perspectives. However, these studies have generally addressed these dimensions separately, focusing either on technological affordances, ethical concerns, assessment challenges, or pedagogical implications. Less attention has been paid to the specific intersection between generative AI and quality assurance in education as an integrated field of inquiry. In particular, there remains a need for a review that combines bibliometric mapping with thematic analysis to identify how quality assurance is being conceptualized, which methodological approaches predominate, and what research gaps emerge in relation to assessment, governance, academic integrity, and educational quality.

Therefore, the present study aims to analyze the scientific production on quality assurance in educational environments mediated by generative artificial intelligence through a scoping review. Specifically, it seeks to: (i) identify the predominant methodological approaches, (ii) analyze the most commonly used conceptual frameworks in the literature, and (iii) determine the main research gaps and future research lines.

In this way, the study contributes to a better understanding of the field and provides a foundation for the development of more comprehensive approaches that enable the responsible, ethical, and high-quality implementation of generative artificial intelligence in education.

Although the study addresses the education field broadly, it does not assume that generative AI-mediated quality assurance is limited to higher education. The review included studies from different educational levels, including higher education, secondary education, K–12 settings, teacher education, and professional or tertiary training, provided that they explicitly examined generative AI in relation to educational quality, assessment, quality assurance, ethics, governance, or academic integrity. Nevertheless, the analysis revealed that higher education represents the most visible and recurrent context in the current literature.

## 2 Methodology

### 2.1 Study design

The present study was designed as a combined bibliometric and scoping review, following the methodological guidelines proposed by Arksey and O’Malley (2005), later refined by Levac et al. (2010), and reported in accordance with the PRISMA-ScR (Preferred Reporting Items for Systematic Reviews and Meta-Analyses Extension for Scoping Reviews) guideline. This combined approach is particularly suitable for examining emerging, complex, and rapidly evolving research fields because it allows both the quantitative mapping of scientific production and the qualitative identification of conceptual, methodological, and thematic patterns.

The scoping review allows for the systematic mapping of existing scientific production, the identification of research trends, conceptual frameworks, methodological approaches, and gaps in the literature (Vargas-Veliz et al., 2026). Additionally, the bibliometric component enabled a detailed examination of publication trends, citation patterns, indexed sources, international collaboration networks, and keyword co-occurrence structures. Within this framework, the aim of the present study was to analyze the available scientific evidence on quality assurance in education mediated by generative artificial intelligence, with particular emphasis on the challenges, implementation strategies, evaluation frameworks, ethical concerns, and governance implications across different educational levels.

The review process was structured to ensure transparency, reproducibility, and alignment with PRISMA-ScR reporting standards. The review was guided by the following main research question: How has the scientific literature addressed quality assurance in educational environments mediated by generative artificial intelligence in terms of methodological approaches, conceptual frameworks, educational levels, ethical concerns, assessment practices, and research gaps?

Specifically, the review sought to answer the following subquestions: (i) What are the main publication trends and collaboration patterns in the field? (ii) What methodological approaches and conceptual frameworks predominate in the literature? (iii) What educational levels and quality assurance dimensions are most frequently addressed? and (iv) What gaps and future research lines emerge from the literature?

No formal review protocol was registered prior to conducting the study. Nevertheless, the review adhered to a predefined methodological plan that clearly outlined the selected databases, search strategy, eligibility criteria, screening process, data extraction variables, bibliometric procedures, and thematic coding approach. To enhance transparency, a PRISMA-ScR checklist was prepared and included as Supplementary material.

References that did not directly focus on generative AI, education, or quality assurance were included only when they offered methodological support—such as guidance on review design, database selection, bibliometric techniques, or thematic analysis. These sources were not used as evidence for the substantive findings of the review.

### 2.2 Information sources and search strategy

The systematic search was conducted in the Web of Science (WoS) and Scopus databases, selected for their broad multidisciplinary coverage in education, social sciences, technology, and innovation (Vasudevan et al., 2025; Singh et al., 2021). These databases provide access to high-impact scientific literature, ensuring the quality and relevance of the analyzed corpus.

The search was conducted on February 15, 2026. The time frame 2022–2026 was selected because the public release and rapid dissemination of generative AI tools, particularly ChatGPT and GPT-based systems, intensified scholarly debate on their implications for education, assessment, academic integrity, and quality assurance. Although 2026 was an incomplete year at the time of data collection, records from this year were included to capture the most recent developments in this rapidly evolving field. The language restriction to English and Spanish was applied because these languages were accessible to the review team and allowed the inclusion of both international and Ibero-American scientific production.

The search strategy was designed by combining key terms associated with three fundamental dimensions: (i) generative artificial intelligence, (ii) educational contexts, and (iii) quality assurance. The search equations were applied in the title, abstract, and keyword fields, adapting the syntax to each database.

In Web of Science, the TS (Topic) field was used, while in Scopus the TITLE-ABS-KEY field was employed. The equations were as follows:

TITLE-ABS-KEY [(“generative AI” OR “large language model*” OR “LLM*” OR “ChatGPT” OR “GPT-4” OR “AI-generated content”) AND (“education” OR “higher education” OR “teaching” OR “learning” OR “academic context”) AND (“quality assurance” OR “quality management” OR “educational quality” OR “quality evaluation” OR “academic quality” OR “standards” OR “accreditation” OR “quality framework*” OR “quality control”)].

TS = [(“generative AI” OR “large language model*” OR “LLM*” OR “ChatGPT” OR “GPT-4” OR “AI-generated content”) AND (“education” OR “higher education” OR “teaching” OR “learning” OR “academic context”) AND (“quality assurance” OR “quality management” OR “educational quality” OR “quality evaluation” OR “academic quality” OR “standards” OR “accreditation” OR “quality framework*” OR “quality control”)].

Filters were applied to include only articles and reviews published in English or Spanish during the period 2022–2026. This time interval is justified by the recent development of generative AI models and their rapid adoption in the educational field, particularly following the dissemination of tools such as ChatGPT.

### 2.3 Eligibility criteria and corpus validation

Eligibility criteria were established to ensure that the final corpus focused specifically on the intersection of three core dimensions: generative artificial intelligence, education, and quality assurance. For this review, generative artificial intelligence was defined as AI systems capable of creating new textual, visual, audio, code-based, or multimodal content. This included large language models, AI chatbots, GPT-based tools, AI-generated content systems, and other generative technologies explicitly applied in educational settings. Studies that focused solely on traditional, rule-based, predictive, or non-generative AI systems were excluded unless they directly addressed generative AI applications.

Education was operationalized as formal or professional learning contexts, encompassing higher education, secondary education, K–12 schooling, teacher training, vocational programs, and other tertiary or professional development settings. Studies were included only if they explicitly examined the use, implementation, evaluation, or implications of generative AI in relation to teaching, learning, assessment, curriculum development, academic writing, instructional design, student support, teacher practices, or broader institutional educational processes.

Quality assurance was defined as the range of processes, standards, policies, evaluation mechanisms, institutional practices, and governance arrangements designed to ensure the reliability, validity, transparency, equity, academic integrity, ethical use, and overall educational value of generative AI-mediated teaching, learning, assessment, and decision-making. Accordingly, the review was not limited to formal accreditation systems; it also encompassed related aspects such as educational quality, assessment validity, quality control, academic integrity, ethical governance, institutional policy, and responsible AI use in education.

To ensure the robustness of the corpus, each record was evaluated using a three-dimensional inclusion framework. A study was included only if it satisfied all three conditions: (i) it explicitly addressed generative AI or closely related tools (e.g., ChatGPT, GPT-based systems, large language models, AI chatbots, or AI-generated content); (ii) it was situated within an educational or learning-related context; and (iii) it engaged with at least one quality assurance dimension, such as assessment integrity, educational quality, reliability, validity, transparency, ethical governance, institutional policy, accreditation, quality standards, or responsible implementation.

Studies were excluded if they discussed artificial intelligence in general without a clear focus on its generative applications, if they examined generative AI outside educational contexts, or if they addressed education and AI without linking to any quality assurance-related concerns. Similarly, works centered exclusively on technical model development, computational performance, or non-educational AI applications were omitted unless they explicitly connected these elements to educational quality, assessment, governance, or reliability in learning environments.

The validation process involved two stages. First, titles, abstracts, and keywords were screened using predefined terms associated with generative AI, education, and quality assurance. Second, pre-selected records underwent manual full-text review when necessary to confirm their alignment with the review’s scope. This rigorous procedure ensured that the final corpus consisted of studies meaningfully positioned at the intersection of generative AI, education, and quality assurance considerations.

### 2.4 Study selection process

Records retrieved from Web of Science and Scopus were exported in .xlsx and .csv formats, respectively, and integrated into a single dataset for processing in RStudio. In the first stage, identification and removal of duplicates were performed using the DOI as the primary identifier and, complementarily, the normalized lowercase title matching.

Subsequently, a preliminary semi-automated screening was conducted using the grepl() function in R, employing key terms related to the phenomenon under study, such as “generative AI,” “ChatGPT,” “quality assurance,” “education,” and “evaluation.” This filtering helped reduce the number of records and focus the analysis on those potentially relevant.

In the second phase, a manual review of titles and abstracts was carried out to verify the alignment of the studies with the established inclusion criteria. Finally, the pre-selected documents were evaluated for definitive eligibility. Full texts were consulted when the information available in titles, abstracts, and keywords was insufficient to determine whether a study met the inclusion criteria. This procedure ensured that the final corpus was aligned with the objective of mapping generative AI-mediated quality assurance in education.

The screening process was conducted by two reviewers. First, both reviewers independently assessed titles and abstracts according to the inclusion and exclusion criteria. Disagreements were discussed until consensus was reached. When consensus could not be achieved, a third reviewer was consulted to make the final decision. This procedure was applied to improve consistency in the selection of studies and reduce selection bias.

**Figure 1:**
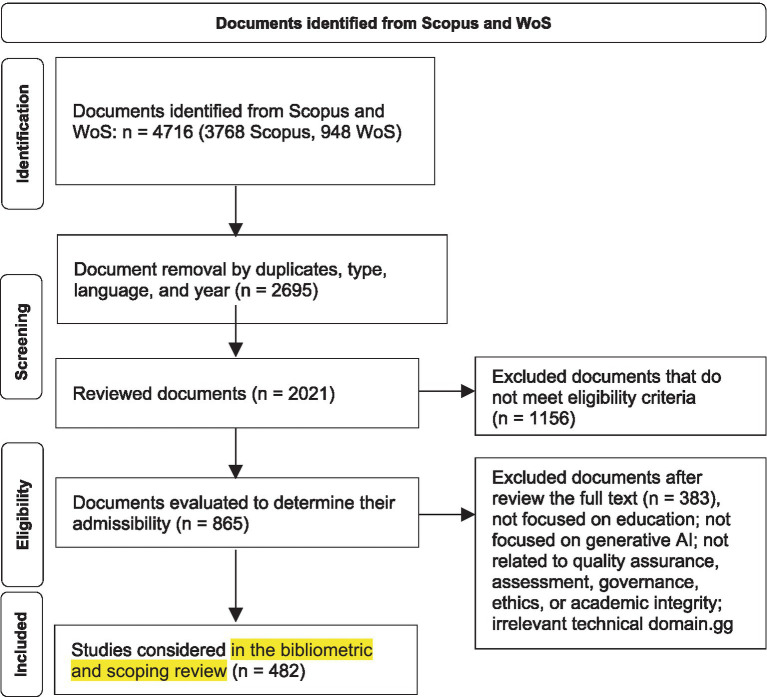
Flow diagram PRISMA.

The selection process was structured according to the PRISMA scheme. In the identification phase, a total of 4,716 records were retrieved. After filtering documents by language, year, and document type, 1935 records remained in Scopus and 747 in WoS. Following the removal of duplicates (661), a set of 2021 records was obtained. During the screening stage, 1,156 studies were excluded for not meeting the thematic criteria, leaving 482 documents for the final corpus of studies included in the bibliometric and scoping review (see Figure 1).

### 2.5 Data extraction

From the included studies, a systematic process of extracting relevant information for analysis was carried out. The variables considered included the year of publication, the country or region of study, the educational level addressed, the type of generative AI tool used, the research objectives, the methodological approach, the identified quality assurance frameworks, the evaluated dimensions (such as quality, reliability, validity, and ethics), the implemented institutional strategies, and the main findings reported.

### 2.6 Analysis strategy

Consistent with the bibliometric and scoping review design, the analysis combined descriptive bibliometric techniques with qualitative thematic analysis. First, a descriptive bibliometric analysis was conducted to explore trends in annual scientific production, citation patterns, leading journals, international collaboration networks, and keyword co-occurrence. Bibliographic metadata from Scopus and Web of Science were merged and cleaned in RStudio and then imported into VOSviewer for network visualization. The bibliometric analysis focused on two primary network structures: country co-authorship and keyword co-occurrence.

For the VOSviewer analyses, the full counting method was used, assigning equal weight to each co-authorship or keyword occurrence instead of applying fractional counting. Country co-authorship networks were constructed using author affiliation data, with a minimum threshold of one document per country to include both established and emerging contributors. Only countries meeting this threshold and showing at least one collaboration link were retained in the final visualization.

Keyword co-occurrence analysis was performed using both author-provided and indexed keywords. A minimum threshold of five occurrences was applied to ensure sufficient relevance. Keywords meeting this threshold and connected to at least one other term were included in the map. Prior to visualization the keyword list was manually reviewed and standardized to address spelling variations singular/plural forms and closely related terms

The association strength normalization method was applied to construct the networks, following common practice in VOSviewer for adjusting link strength based on relationship intensity. Clusters were identified using VOSviewer’s default clustering algorithm. In the resulting visualizations, node size reflected frequency or productivity, link thickness indicated relationship strength, and colors denoted thematic clusters. Only items meeting the established thresholds and showing connections were included in the final maps.

Different tools were employed according to the needs of each analytical stage. RStudio was used for data integration, cleaning, duplicate removal, title standardization, and initial keyword-based screening [via functions such as grepl()]. VOSviewer was dedicated exclusively to bibliometric visualization, particularly for generating country co-authorship and keyword co-occurrence networks. The thematic analysis, in contrast, was performed manually through repeated reading and coding of titles, abstracts, keywords, and bibliographic metadata, without the use of automated qualitative analysis software.

Second, a qualitative thematic analysis was carried out on the titles, abstracts, keywords, and bibliographic metadata of the 482 included studies. Full texts were consulted only during the eligibility screening phase when additional information was required. This approach was appropriate for a bibliometric and scoping review, whose main goal was to map predominant methodological approaches, conceptual frameworks, quality assurance dimensions, and emerging research lines. Accordingly, the thematic findings should be viewed as an exploratory mapping rather than an exhaustive full-text synthesis.

The thematic analysis followed the six-phase procedure outlined by Braun and Clarke (2006). In the first phase, titles, abstracts, keywords, and metadata were read and re-read to gain familiarity with the corpus and note initial impressions. In the second phase, initial codes were generated by identifying recurring concepts related to generative AI, educational quality, assessment, reliability, validity, transparency, ethics, academic integrity, pedagogical innovation, institutional governance, and responsible AI use. In the third phase, related codes were organized into preliminary categories. The fourth phase involved reviewing these categories against both the coded extracts and the full set of abstracts to ensure coherence and consistency. In the fifth phase, themes were refined, clearly defined, and named according to their relevance to the research questions. Finally, in the sixth phase, the themes were synthesized into a coherent narrative and discussed in relation to the bibliometric findings and existing literature.

The coding framework was developed inductively and refined iteratively throughout the process. Initial codes were grouped into broader categories such as methodological approach, educational level, type of generative AI tool, quality assurance dimension, assessment concerns, ethical or governance issues, pedagogical implications, and research gaps. These categories served as an analytical guide rather than rigid templates, helping to organize the data and identify recurrent research lines.

To strengthen coding reliability, two reviewers independently coded an initial subset of records. They then compared results, discussed discrepancies, and refined the framework before applying it to the full corpus. Disagreements were resolved through discussion and consensus; when needed, a third reviewer was consulted. This iterative process of comparison and refinement helped ensure consistency between codes, emerging themes, and the overall research questions.

Through comparison, refinement, and consolidation of codes, four major thematic lines emerged: (i) challenges associated with generative AI in educational quality assurance, (ii) institutional and pedagogical implementation strategies, (iii) evaluation frameworks and quality models, and (iv) ethical, regulatory, and governance implications. These themes were not predetermined but arose naturally from the iterative analysis of the abstracts and were supported by the keyword co-occurrence patterns observed in VOSviewer.

Because the thematic analysis relied primarily on titles, abstracts, keywords, and bibliographic metadata, the findings represent an exploratory mapping of the field. Consequently, they should be interpreted as an overview of dominant orientations, recurring concerns, and emerging directions rather than a comprehensive conceptual synthesis based on full-text analysis of all studies.

### 2.7 Methodological quality considerations

In accordance with the purpose of a scoping review, no formal risk-of-bias or methodological quality appraisal was conducted as an exclusion criterion. The objective of this study was not to evaluate intervention effectiveness or establish causal relationships, but to map the extent, structure, methodological orientations, conceptual frameworks, and research gaps of an emerging field. Nevertheless, the analysis considered the reported study design, methodological approach, and type of evidence when interpreting the maturity and development of the literature.

## 3 Results

### 3.1 Temporal evolution of scientific production and citation impact

**Figure 2:**
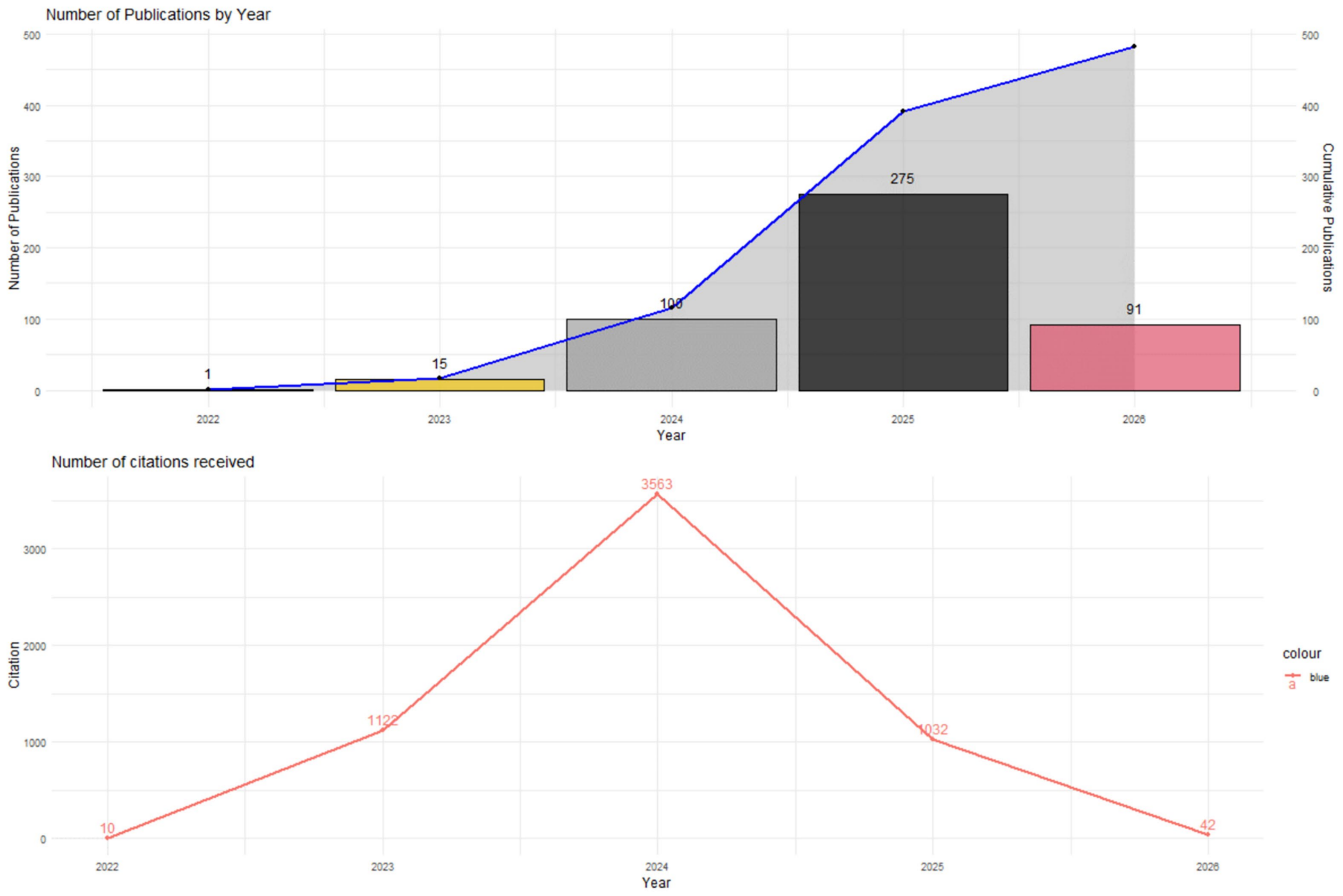
Annual evolution of publications and citations in studies on generative AI and quality assurance in education (2022–2026).

Figure 2 presents the temporal evolution of both scientific production and impact measured through the number of citations in the field of quality assurance in education mediated by generative artificial intelligence during the period 2022–2026.

Citation data were obtained from the bibliographic metadata exported from Scopus and Web of Science on February 15, 2026. Therefore, citation counts should be interpreted as time-sensitive indicators that reflect the status of the databases at the date of extraction.

First, an exponential growth trend in the number of publications is observed, particularly from 2024 onward. While in 2022 the production was practically incipient (1 publication), in 2023 a moderate increase was recorded (15 publications), followed by substantial growth in 2024 (100 publications). This trend intensified in 2025, reaching its peak with 275 publications, evidencing a consolidation of the field of study. Although a decrease is observed in 2026 (91 publications), this can be explained by the incomplete nature of the current year rather than a real reduction in research interest.

In terms of impact, the citation behavior shows a different dynamic. A significant peak is identified in 2024 (3,563 citations), suggesting that studies published that year have had high visibility and influence in the scientific community. This phenomenon may be associated with the initial boom of tools such as ChatGPT and the global interest in understanding their implications in educational contexts. Subsequently, a decrease is observed in 2025 (1,032 citations) and 2026 (42 citations), which is consistent with the citation window effect, as the most recent articles have not yet had sufficient time to accumulate citations.

The joint analysis of both trends reveals a pattern characteristic of emerging fields: accelerated expansion in scientific production accompanied by a concentration of impact in the early years of development. This suggests that the area of study is in a phase of rapid consolidation, where the volume of research grows steadily, but academic recognition is still unevenly distributed between pioneering and more recent works.

Taken together, these results demonstrate that quality assurance in education with generative AI constitutes an emerging field with high growth dynamics, driven by recent technological advances and a growing concern for the reliability, validity, and governance of these tools in educational environments.

### 3.2 Distribution of scientific production and its impact by indexed sources

Table 1 presents the distribution of scientific production and its impact, measured through the number of citations, in the main journals that have published research on generative artificial intelligence applied to quality assurance in education.

**Table 1:** Main scientific journals according to number of publications and citations in studies on generative AI and quality assurance in education.

Source title	Quantity	Citations	Citations per paper
IEEE access	20	52	2.60
Information (Switzerland)	8	162	20.25
Applied sciences (Switzerland)	8	6	0.75
Journal of applied learning and teaching	6	225	37.50
Discover education	6	19	3.17
Interactive learning environments	5	540	108.00
Education sciences	5	104	20.80
IEEE transactions on learning technologies	5	86	17.20
Education and information technologies	5	72	14.40
Electronics (Switzerland)	5	26	5.20

In terms of productivity, the IEEE Access journal stands out as the most relevant source, with a total of 20 publications, reflecting its role as a key platform for the dissemination of interdisciplinary research in technology and education. However, its impact in terms of citations (52) is relatively moderate compared to other journals, suggesting that a higher number of publications does not necessarily translate into greater academic influence.

On the other hand, journals such as Interactive Learning Environments stand out for their high impact, accumulating 540 citations with only 5 publications. This pattern indicates a high citation density, suggesting that the articles published in this source possess strong theoretical and empirical relevance within the field. Similarly, the Journal of Applied Learning and Teaching shows a prominent performance, with 225 citations distributed across 6 documents, consolidating itself as an influential space in the pedagogical discussion of emerging technologies.

Furthermore, the presence of hybrid journals that integrate technological and educational approaches, such as Education and Information Technologies and IEEE Transactions on Learning Technologies, is observed. These show a balance between production (5 publications each) and impact (72 and 86 citations, respectively). This result evidences the interdisciplinary nature of the field, where engineering, computer science, and education sciences converge.

In contrast, some journals such as Applied Sciences (Switzerland) and Electronics (Switzerland) present relatively low citation levels in relation to their production, which may indicate that the studies published in these sources are more oriented toward technical applications than toward pedagogical or quality assurance debates, thus limiting their visibility in the educational literature.

Overall, the results reveal a dissociation between productivity and impact, as well as a clear trend toward the concentration of impact in journals specialized in education and learning technologies. This suggests that, although the field is being addressed from multiple disciplines, the most influential contributions are found in those journals that explicitly articulate the relationship between technology and educational processes.

### 3.3 International scientific collaboration networks in research on generative AI in education

**Figure 3:**
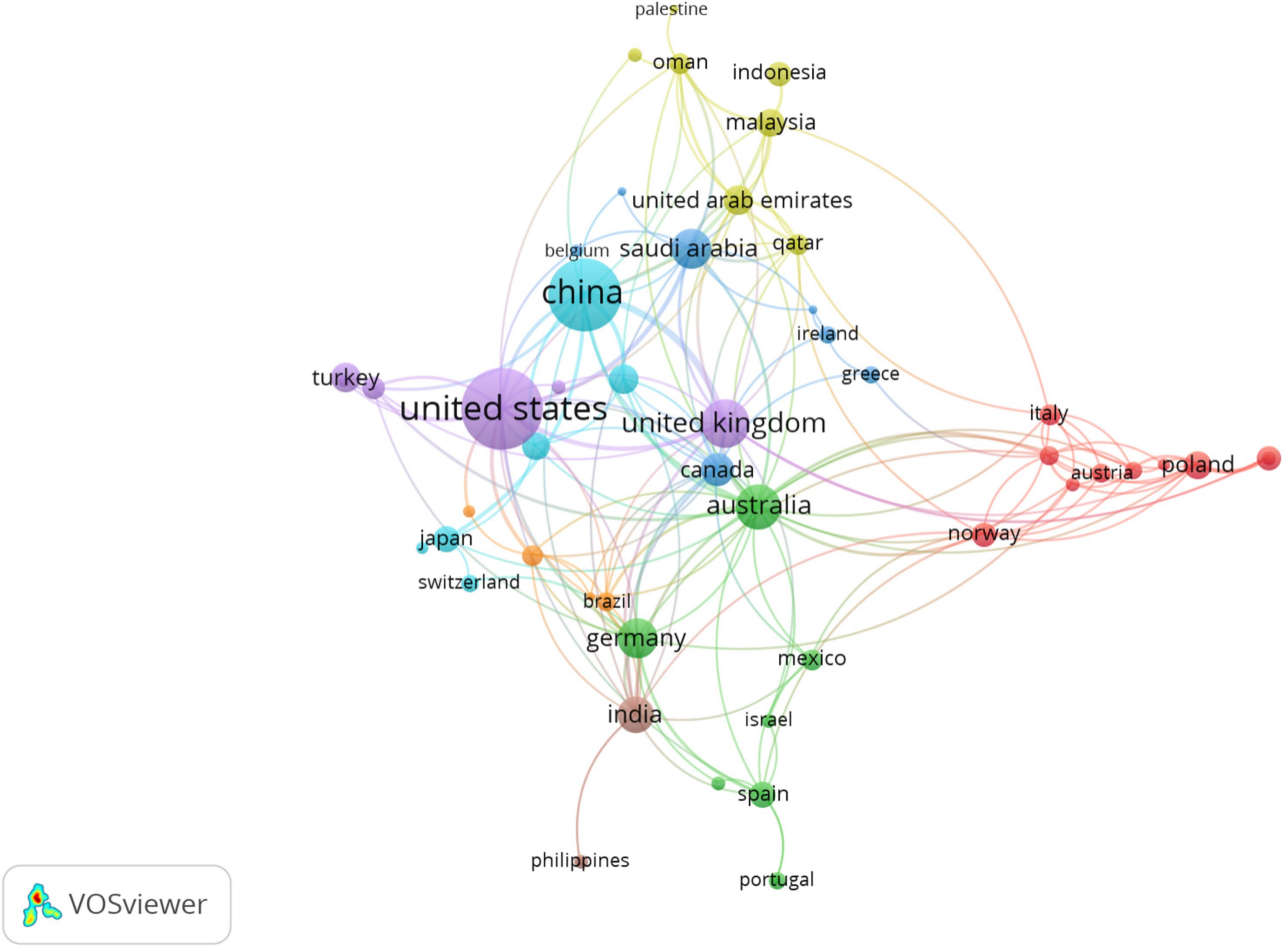
Co-authorship network between countries in studies on generative AI and quality assurance in education.

International scientific collaboration constitutes a key dimension for understanding the development of research on generative artificial intelligence applied to quality assurance in education. In this study, the co-authorship network between countries was analyzed to identify the main collaboration hubs, regional clusters, and peripheral participation patterns. As shown in Figure 3, the network reveals the existence of multiple collaboration clusters, differentiated by colors, which group countries with more frequent co-authorship links.

At the center of the network are countries such as the United States, the United Kingdom, and China, which act as highly connected nodes, playing a key role as global collaboration hubs. In particular, the United States stands out for its broad connectivity with various regions, reflecting its leadership in both scientific production and the articulation of international networks. Similarly, the United Kingdom and China show strong integration in the network, connecting with both developed countries and emerging economies.

Well-defined regional clusters are identified. For example, a European cluster composed of countries such as Italy, Austria, Poland, and Norway presents a high density of internal connections, suggesting consolidated collaboration within the region. Likewise, a cluster led by Australia, Germany, and Spain acts as a bridge between Europe and other regions, including Latin America and Asia.

In the Asian and Middle Eastern context, countries such as Saudi Arabia, the United Arab Emirates, Malaysia, and Indonesia form an emerging cluster with growing participation in the global network. This pattern indicates a geographical expansion of the field of study, driven by recent investments in educational technologies and digital transformation.

On the other hand, countries such as Mexico, Brazil, and India appear as peripheral but strategic nodes, connecting different clusters and evidencing expanding participation, although still limited in terms of centrality. Likewise, the presence of countries with fewer connections, such as Palestine or Oman, suggests an incipient participation in global scientific production.

Overall, the network reveals a highly interconnected but hierarchical structure, where a small group of countries leads scientific production and coordination, while others participate more peripherally. This pattern is characteristic of emerging fields, in which international collaboration plays a fundamental role in the consolidation of knowledge.

These findings highlight the need to foster greater inclusion of underrepresented regions, particularly Latin America and Africa, in order to promote more equitable and contextualized scientific production in the field of quality assurance in education mediated by artificial intelligence.

### 3.4 Thematic structure and research trends in generative AI applied to educational quality

**Figure 4:**
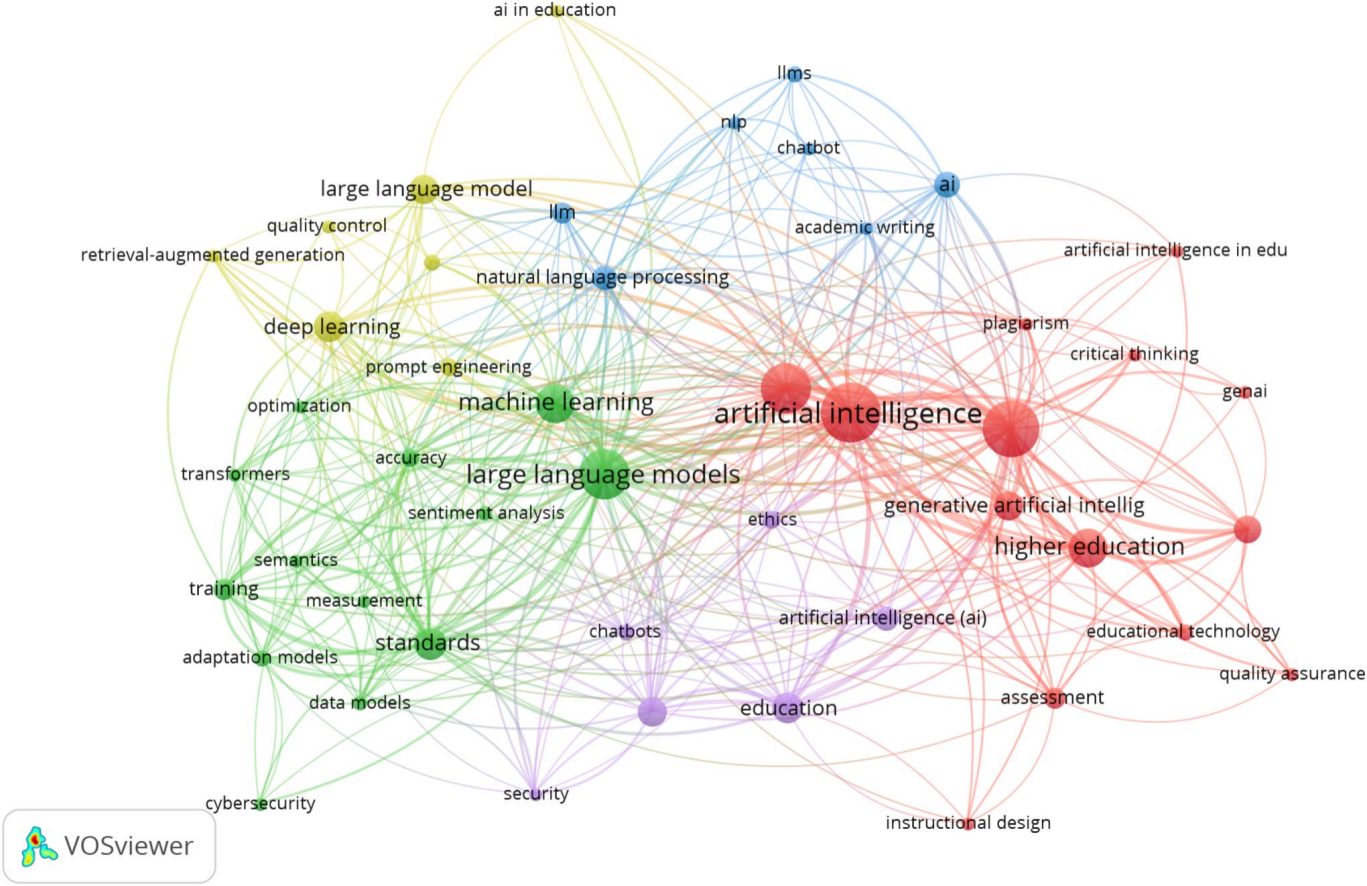
Keyword co-occurrence map in studies on generative AI and quality assurance in education.

Figure 4 presents the keyword co-occurrence map generated using VOSviewer, which allows identification of the conceptual structure of the field of study, as well as the main emerging thematic lines in research on generative artificial intelligence applied to quality assurance in education.

The analysis reveals the presence of multiple well-defined clusters, evidencing the multidimensional and interdisciplinary nature of the field. At the core of the network are highly connected terms such as “artificial intelligence,” “large language models,” and “higher education,” which act as articulating axes of knowledge. The centrality of these terms indicates that most research focuses on the integration of large-scale language models in higher education contexts.

The cluster represented in red groups concepts related to educational implementation and assessment, including terms such as “assessment,” “educational technology,” “quality assurance,” “plagiarism,” and “critical thinking.” This thematic set reflects a growing concern about the effects of generative AI on academic assessment processes, educational integrity, and the quality of learning, positioning itself as one of the most relevant lines in the field.

The green cluster is associated with the technical foundations of artificial intelligence, with terms such as “machine learning,” “deep learning,” “transformers,” “prompt engineering,” and “accuracy.” This group evidences that a significant part of the literature maintains a technical focus, oriented toward the development and optimization of models, suggesting a strong influence of disciplines such as computer science and engineering in the study of the phenomenon.

The blue cluster, which includes terms such as “chatbot,” “NLP,” “academic writing,” and “LLMs,” focuses on specific applications of generative AI in educational environments, particularly in the production of academic content and automated interaction with students. This approach highlights the role of tools such as ChatGPT in the transformation of traditional educational practices.

The yellow cluster incorporates terms such as “AI in education,” “large language model,” “quality control,” and “retrieval-augmented generation,” indicating an emerging line focused on the integration of advanced technologies to improve the quality and reliability of AI-based educational systems.

Finally, the purple cluster groups concepts linked to education as a system, including “education,” “ethics,” “security,” and “chatbots,” highlighting concerns related to governance, ethics, and security in the use of these technologies.

Overall, the map shows that the field of study is structured around three major dimensions: (i) technological development of AI models, (ii) educational and pedagogical applications, and (iii) evaluation, quality, and ethical considerations. This configuration suggests an evolution from technical approaches toward a more critical and applied integration in the educational field, where quality assurance emerges as a transversal axis.

These findings confirm that research on generative AI in education is not only expanding but is also undergoing a process of thematic diversification and conceptual consolidation, which opens opportunities for the development of comprehensive quality assessment frameworks adapted to educational environments mediated by artificial intelligence.

### 3.5 Methodological approaches and conceptual frameworks

Before interpreting these findings, it is important to note that the classification of methodological approaches and conceptual frameworks was based primarily on information available in titles, abstracts, keywords, and bibliographic metadata. Full texts were consulted only when the information available at this level was insufficient to determine eligibility. Consequently, the results presented in this section should be interpreted as an exploratory mapping of visible trends in the corpus rather than as a comprehensive full-text synthesis of the conceptual foundations of each study.

Based on the thematic coding of the abstracts, four major analytical dimensions were identified: methodological approaches, quality and assessment frameworks, ethical and governance concerns, and pedagogical or academic integrity-related implications. These dimensions emerged from the iterative coding process and were subsequently contrasted with the keyword co-occurrence map to verify their conceptual consistency within the broader structure of the field.

The predominant methodological approaches and conceptual frameworks used in the literature on quality assurance in educational environments mediated by generative artificial intelligence were identified based on the thematic analysis of the abstracts of the 482 included studies.

The categories used to classify methodological approaches and conceptual frameworks were not strictly mutually exclusive. A single article could be assigned to more than one conceptual category when it addressed multiple dimensions, such as assessment, ethics, governance, and academic integrity. However, methodological classification was based on the dominant design reported in the title, abstract, keywords, and, when necessary, full-text consultation during eligibility checking.

Regarding methodological approaches, the abstract-level mapping suggests a predominance of review studies, with a total of 124 documents classified as systematic reviews, scoping reviews, or bibliometric analyses. The predominance of review studies is consistent with works oriented toward synthesizing quality criteria, assessment, and use of AI in education, such as the study by Sarker and Ullah (2023) on quality assessment criteria in secondary education and the integrative review by Chaka (2024) on tools for detecting texts generated by AI. These antecedents suggest that the field is still in a stage of conceptual organization and delimitation of analytical frameworks.

On the other hand, empirical studies with a quantitative approach also show significant presence (190 documents), including research based on surveys, statistical models, and techniques such as regression or structural equations. In this line, Yang et al. (2025) analyzed, through a mixed-methods approach, students’ perspectives on the ethics of generative AI, while Cheng et al. (2025) developed a mixed-methods study on the implementation of generative AI in higher education programs. Likewise, Ahangama (2026) proposed an evaluation framework adapted to the era of generative AI for tertiary ICT education, evidencing an applied orientation toward the redesign of academic assessment. Additionally, 83 experimental studies were identified, reflecting a growing interest in controlled evaluation of the impact of generative AI on educational processes. To a lesser extent, 85 qualitative studies were found, based on interviews, case studies, and thematic analysis, suggesting a still limited in-depth exploration of educational experiences mediated by these technologies.

Regarding conceptual frameworks, the thematic mapping indicates that one of the most visible dimensions corresponds to quality and educational assessment, present in 218 studies. This dimension can be linked to works focused on fairer and AI-adapted assessment practices, such as the conceptual article by Dollinger and Nieminen (2026), which rethinks notions of success, failure, and equitable assessment in higher education. Complementarily, Elkhodr and Gide (2025) proposed the SAGE framework to promote critical thinking and responsible use of generative AI in cybersecurity education, providing a concrete reference for the pedagogical and formative integration of these tools.

Second, there is a strong presence of studies focused on artificial intelligence ethics (167 documents), addressing issues such as governance, academic integrity, and responsible use. In particular, Yang et al. (2025) explored students’ perceptions of generative AI ethics; Thaldar et al. (2025) analyzed institutional governance of generative AI in higher education from an African case study; and Artyukhov et al. (2024) examined the relationship between artificial intelligence and academic integrity within the framework of SDG 4. Together, these works show that ethical concern is not marginal but one of the most visible lines in recent literature.

To a lesser extent, pedagogical frameworks appear less frequently in the abstracts and metadata analyzed are identified (68 studies), related to teaching-learning processes, educational innovation, and curricular adaptation, which is consistent with recent research analyzing the integration of generative AI in pedagogical practices and instructional design (Cheng et al., 2025; Elkhodr and Gide, 2025). Finally, the dimension of academic integrity appears marginally, with very limited presence, suggesting an area that is still underexplored despite its relevance in the context of tools such as ChatGPT, as has been pointed out in recent studies on ethics and integrity in AI-mediated educational environments (Artyukhov et al., 2024; Chaka, 2024).

Overall, these results suggest that the visible literature in this field is characterized by a strong orientation toward quality assessment and ethical implications, accompanied by an expanding methodological diversity. Likewise, a significant opportunity is identified for the development of more robust methodological approaches and integrated conceptual frameworks that articulate the pedagogical, technological, and governance dimensions in a more balanced manner.

## 4 Discussion

The evidence obtained confirms that quality assurance in educational environments mediated by generative artificial intelligence is configured as an emerging field characterized by rapid conceptual expansion, but with methodological consolidation still under development. This pattern has been widely documented in recent literature, where a proliferation of studies oriented toward mapping the phenomenon rather than modeling it empirically is observed (Bearman and Ajjawi, 2023; Jukiewicz, 2024; Yan et al., 2024).

It is important to distinguish quality assurance from related but non-equivalent concepts. Quality assurance refers to the broader set of institutional, pedagogical, technical, and ethical processes aimed at ensuring that AI-mediated educational practices meet expected standards of reliability, validity, transparency, equity, and educational value. Quality assessment focuses more specifically on measuring learning outcomes, performance, or the quality of educational products and processes. Quality control refers to mechanisms used to detect and correct errors or deviations from established standards. Evaluation involves the systematic judgment of programs, tools, or interventions, while accreditation refers to formal external recognition based on institutional or program-level standards. Academic governance encompasses the policies, responsibilities, and decision-making structures that regulate the use of generative AI in educational institutions.

The predominance of review studies identified in the results aligns with what has been pointed out by Bouteraa et al. (2024), who highlight that research on generative artificial intelligence in education has initially evolved as a process of systematization of knowledge. This behavior is characteristic of disruptive fields, where the speed of technological adoption surpasses the capacity to generate consolidated empirical evidence. In this sense, the high presence of reviews not only reflects an exploratory phase but also an effort to establish common interpretive frameworks (Popenici et al., 2023; Zaim et al., 2024).

Nevertheless, the growing presence of quantitative and experimental studies evidences a transition toward more robust approaches oriented toward empirical validation. Research such as that by Hu et al. (2024) demonstrates the use of statistical models, performance analysis, and experimental designs to evaluate the impact of AI-based tools on learning. This methodological advance suggests a maturation process of the field, although still coexisting with descriptive and exploratory approaches (Alshurafat et al., 2024).

From a conceptual perspective, the centrality of quality and educational assessment confirms that the academic debate has been structured primarily around the measurement of learning and the validity of results generated through AI. Studies such as those by Ali et al. (2024), Feretzakis et al. (2024), and Qadhi et al. (2024) agree in pointing out that the irruption of tools such as ChatGPT has strained traditional evaluation systems, questioning criteria such as authenticity, originality, and reliability. In the same line, Head et al. (2023) and Hung and Chen (2023) warn that educational systems still lack standardized frameworks that allow the effective integration of these technologies into assessment processes.

In parallel, the strong presence of the ethical dimension evidences that quality assurance in this context cannot be separated from the principles of technological governance. Recent research highlights concerns related to algorithmic bias, transparency, and accountability in the use of generative systems (Aljuaid, 2024; Alshurafat et al., 2024). This emphasis is also reinforced by emerging studies that propose regulatory frameworks and institutional policies for the responsible use of AI in education (Azevedo et al., 2024; Flenady and Sparrow, 2025; Qian et al., 2026).

However, the lower presence of pedagogical frameworks suggests a disconnection between technological development and its didactic integration. Although research such as that by Gamage et al. (2023), Chaka (2024), and Filiz et al. (2025) addresses the potential of generative AI in teaching-learning processes, these approaches are still incipient compared to the evaluative and ethical dimensions. This gap evidences that the academic discussion has prioritized risks and performance measurement over pedagogical redesign.

Likewise, the limited attention to academic integrity is particularly critical. Studies such as those by Ray (2023), and Desaire et al. (2023) warn that the use of generative artificial intelligence poses significant challenges in terms of plagiarism, authorship, and automated academic production. Despite this, the low representation of this dimension in the literature suggests that a solid theoretical body has not yet been developed to address these issues in an integral manner.

On the other hand, the most recent studies (2026) introduce new research lines oriented toward technological infrastructure, system reliability, and the integration of advanced models such as retrieval-augmented generation and autonomous systems (Dollinger and Nieminen, 2026; Jiang et al., 2026). Although these approaches expand the field of study, they also reinforce the need to articulate these developments with educational and quality frameworks, avoiding fragmentation between the technological and the pedagogical.

### 4.1 Toward a multi-level framework for quality assurance in generative AI-mediated education

Beyond the descriptive bibliometric patterns, the findings highlight the need to conceptualize quality assurance in generative AI-mediated education as a multi-level and multi-layered process. This approach moves the field beyond isolated discussions of assessment, ethics, or technological performance toward a more integrated understanding of how educational quality can be maintained when generative AI is embedded in teaching, learning, and institutional decision-making.

At the learner level, quality assurance focuses on the impact of generative AI on learning outcomes, cognitive engagement, student agency, feedback quality, authorship, originality, and responsible academic behavior. Here, quality is understood not only in terms of the accuracy of AI-generated outputs, but also in whether students develop critical thinking skills, remain actively involved in their learning, and use AI tools to support rather than replace their own cognitive processes. This level is especially important because generative AI is transforming how students search for information, produce academic texts, solve problems, seek feedback, and make decisions about their learning.

At the teaching and assessment level, quality assurance calls for the redesign of pedagogical practices and evaluation systems. Generative AI challenges traditional notions of originality, authorship, assessment validity, and student performance. Therefore, teaching practices should include clear guidelines for the responsible use of AI, while assessment strategies need to emphasize authenticity, transparency, process-oriented evaluation, critical reflection, and tasks that make students’ reasoning visible. In this context, quality assurance involves not only detecting misuse but also redesigning learning activities so that AI integration becomes pedagogically meaningful and ethically grounded.

At the institutional governance level, quality assurance relies on policies, standards, accountability mechanisms, professional development, and decision-making structures. Educational institutions require clear guidelines on acceptable and unacceptable uses of generative AI, data protection, academic integrity, teacher responsibilities, student rights, and the documentation of AI-assisted work. Strong institutional governance is essential for aligning AI adoption with accreditation requirements, curriculum standards, and broader educational goals. Without this level, the integration of generative AI risks remaining fragmented and overly dependent on individual teachers’ decisions.

Technical reliability serves as a transversal layer across the framework. Generative AI introduces risks such as hallucinations, biased outputs, limited explainability, data privacy concerns, model opacity, and performance variability. Quality assurance must therefore incorporate mechanisms to verify the reliability, transparency, traceability, and suitability of AI-generated content. This requires educators and institutions to critically evaluate both the affordances and the inherent limitations of these systems within learning and assessment contexts.

Ethical considerations form a second transversal layer. Quality assurance in generative AI-mediated education must address issues of fairness, bias, accountability, transparency, academic integrity, responsible use, and the protection of learners’ rights. Ethical quality should not be viewed as separate from educational quality; rather, it is a fundamental condition for ensuring that AI-mediated education remains equitable, trustworthy, and centered on human learning. This perspective evaluates the implementation of generative AI not solely by its efficiency or innovation, but by its broader implications for students, teachers, institutions, and educational justice.

Taken together, these levels and layers point to an integrated socio-technical framework for quality assurance in generative AI-mediated education. It connects learner experiences, teaching and assessment practices, institutional governance, technical reliability, and ethical responsibility. This conceptualization advances the field by demonstrating that quality assurance cannot be reduced to plagiarism detection, output verification, or technological optimization. Instead, it requires coordinated efforts across pedagogy, assessment, governance, technology, and ethics to support meaningful human learning in AI-rich educational environments.

Overall, the evidence mapped in this review indicates that the field is shifting from fragmented discussions on assessment, ethics, academic integrity, and technological performance toward a more holistic understanding of quality assurance. The central conceptual contribution of this study is the proposal of a multi-level and multi-layered approach involving learner experiences, pedagogical and assessment redesign, institutional governance, technical reliability, and ethical responsibility. This framework can inform future empirical research, institutional policies, and pedagogical models aimed at ensuring that generative AI enhances meaningful, fair, transparent, and responsible human learning.

### 4.2 Implications for human learning and academic behavior

The findings also carry important implications for human learning and academic behavior in AI-mediated educational settings. At the student level, generative AI can enhance learning by offering immediate feedback, explanations, examples, writing support, and opportunities for self-regulated learning. However, these advantages depend on students using AI as a tool for deeper cognitive engagement rather than a replacement for their own thinking. From a quality assurance standpoint, the key question is whether interactions with generative AI promote critical thinking, metacognition, conceptual understanding, and learner agency.

Generative AI also influences academic behavior. The ease of generating essays, solutions, summaries, and other academic texts may reshape students’ understanding of authorship, originality, effort, and academic responsibility. This creates risks such as plagiarism, over-reliance on AI, superficial learning, and the outsourcing of cognitive work. Rather than relying solely on detection or punitive measures, quality assurance should promote educational strategies that help students recognize appropriate AI use, document AI-assisted contributions, critically evaluate generated content, and retain ownership of their learning.

Assessment practices are significantly affected, as traditional tasks centered on final written products may no longer adequately capture student learning. The findings point to the need for more authentic, transparent, and process-oriented assessment designs. These could include oral defenses, reflective assignments, portfolios, iterative drafts, in-class activities, problem-based tasks, and activities that require students to explain and critically justify their use of AI. In this way, quality assurance should aim not only to prevent misconduct but also to preserve the validity, fairness, and educational value of assessment.

Teacher decision-making represents another key area. Educators must increasingly determine when AI use is pedagogically appropriate, communicate clear expectations, evaluate AI-assisted work, and adapt activities to maintain meaningful human engagement. This demands ongoing professional development, institutional support, and shared criteria for integrating generative AI. Teachers should not be expected to navigate these changes in isolation; instead, their practices should be guided by comprehensive quality assurance frameworks that link pedagogy, assessment, ethics, and technical considerations.

Finally, institutional responses play a crucial role in shaping academic behavior and safeguarding educational quality. Institutions need well-defined policies that clarify acceptable AI use, establish procedures for academic integrity, guide assessment redesign, protect student data, and support responsible practices among teachers and students. These responses should move beyond simple prohibitions toward governance models that treat generative AI as a socio-technical element influencing learning, behavior, assessment, and institutional accountability. Ultimately, quality assurance in generative AI-mediated education should be viewed as a coordinated effort to ensure that AI strengthens, rather than undermines, authentic human learning.

## 5 Conclusion

This bibliometric and scoping review mapped the scientific production on quality assurance in generative AI-mediated education during the period 2022–2026. Based on 482 documents retrieved from Scopus and Web of Science, the findings show that this is a rapidly expanding field characterized by methodological diversity and a strong concentration on quality assessment and ethical concerns.

The abstract-level thematic mapping suggests that review studies remain highly visible, indicating that the field is still undergoing conceptual organization. At the same time, the presence of quantitative and experimental studies points to an emerging interest in empirical validation. However, pedagogical frameworks, academic integrity, and institutional governance remain less developed compared with assessment and ethics.

These findings should be interpreted as a thematic and bibliometric mapping of the field rather than as an in-depth full-text synthesis of all included studies. Even with this limitation, the review highlights the need for future quality assurance frameworks that integrate pedagogical design, assessment validity, technical reliability, ethical responsibility, and institutional governance in generative AI-mediated education.

### 5.1 Limitations

First, given the broad scope of the review and the size of the corpus, the thematic synthesis was based primarily on titles, abstracts, keywords, and bibliographic metadata. Full texts were consulted during the eligibility stage when additional clarification was required. Therefore, the findings should be interpreted as a thematic and bibliometric mapping of the field rather than as an in-depth qualitative synthesis of the full content of each included study.

Second, a considerable proportion of the analyzed articles do not explicitly report their methodological approach in the abstracts, which introduces a degree of uncertainty in the classification performed. This limitation is characteristic of emerging fields where standardization in scientific reporting has not yet been achieved.

Third, although the review used two major international databases, Scopus and Web of Science, the exclusion of other sources such as ERIC, Dimensions, Google Scholar, or regional databases may have generated coverage bias, particularly regarding studies published in local journals, institutional reports, or non-indexed educational research.

Finally, the bibliometric and scoping review approach, while suitable for mapping broad and heterogeneous fields, does not allow for the evaluation of the methodological quality of the included studies or the establishment of causal relationships between variables, which limits the inferential scope of the results.

### 5.2 Future research lines

Based on the findings obtained, several opportunities for the development of future research are identified.

First, greater emphasis is required on empirical studies that rigorously evaluate the impact of generative artificial intelligence on learning outcomes, using experimental, longitudinal designs and advanced statistical models. This would strengthen the evidence base and advance toward greater methodological maturity of the field.

Second, the development of integrated pedagogical frameworks that guide the use of generative AI in educational contexts is essential. The current literature shows a significant gap in this area, which opens the possibility of designing models that articulate technology with learning theories and didactic strategies.

Third, the ethical and technological governance dimension needs to be deepened through studies that analyze the implementation of institutional policies, regulations, and quality standards in the use of artificial intelligence in education. This includes aspects such as algorithmic transparency, equity, and data protection.

Fourth, future reviews could also integrate quantitative and qualitative methods to enrich the interpretation of the results. In this sense, bibliometric techniques may be complemented with an in-depth qualitative content analysis of a purposive sample of highly relevant articles. Such an approach would allow researchers not only to identify publication trends, collaboration patterns, and keyword structures, but also to examine more deeply the theoretical foundations, methodological designs, and practical implications of the studies included. This integration of quantitative and qualitative review methods may provide a more comprehensive understanding of the development of the field (Moresi et al., 2023).

Likewise, academic integrity is positioned as a critical line of research, particularly in relation to the use of generative tools in content production. Studies are needed that redefine concepts such as authorship, originality, and assessment in AI-mediated environments.

Finally, it is recommended to advance toward interdisciplinary approaches that integrate technological, educational, ethical, and social perspectives, allowing for a more holistic understanding of the phenomenon. This type of approach will be key to designing resilient, innovative educational systems aligned with the challenges of digital transformation.

## Data Availability

The original contributions presented in the study are included in the article/supplementary material, further inquiries can be directed to the corresponding author.
